# Sussex County Hospital

**Published:** 1890-12-20

**Authors:** 


### Sussex County Hospital.

### A SURE CURE FOR ENNUI.

It has been our privilege within the last few months to inspect an increasing number of hospitals, many of them in the provinces. Few occupations could have given greater pleasure or satisfaction than we have received from these visits. How many a man and woman groans at the winter months especially, and wonders what to do to pass the time in the afternoons of many days! How many there are who become subscribers to our hospitals, who give an order to their bankers for the payment of the annual subscription, and who then forget all about the matter. We would suggest to such people that they have not yet got full value for their money. It is all very well to subscribe to a hospital, or to any other charity, but to stop there is to show an absence of appreciation little creditable to the individual. It is an old saying that he who pays the piper shall select the tune. So those who support our hospitals are bound, not only in the interest and welfare of the public and the patients, but in their own interests too, to become familiar with their methods of work, and to strive to possess some small portion at any rate of the spirit which undoubtedly animates the large majority of those who constitute the official staff of these institutions. We have been impressed over and over again with a feeling that a hospital nowadays is a cheerful place, where most people are contented and all are genuinely happy. Even the sick feel that they are safe when they find themselves in a hospital bed; and as to the children, why a children's ward has proved a paradise on earth to many a little sufferer and waif, as all our hospitals can testify. We would say, then, to every governor, "Get full value for your money," and if we are asked how, we would say "By making a point of visiting the hospital with some sort of regularity, and so learn lessons in life, lessons in suffering, and, what is even more important, learn to appreciate the privilege of being able to give, and moved to give to the support of institutions like these." Besides, many of the county hospitals have excellent libraries and museums, containing specimens of value and interest, and the matrons and secretaries are always willing to point out a path in hospital work, helpful alike to those who labour and those who benefit by such labours.

### Our Inspection.

We understand that at Brighton it is not the habit of the Brighton people to visit their hospitals. Having recently inspected the Sussex County, we can assure them that they make a very great mistake, and that the sooner they alter their course of action in this matter the better it will be for themselves, as well as for the noble institution at Kemp Town. We assume that a governor decides to follow our advice, and strives to lose personal *ennui* in hospital work. Let all such, and those who are inclined to laugh at the suggestion, accompany us now through the wards and offices of the Sussex County Hospital. It is Saturday afternoon, a time when the visiting staff does not attend at the hospital. The main work of the day is over, the evening is falling in, and the lights in most of the wards are alight. Around the fireplaces in the spacious wards are to be found convalescent patients seated in comfortable chairs, either reading or sewing, or being read to. All are bright, cheerful, and contented. The nurse is in her room adjacent to the ward, and smiles when asked if she has had good health, seeing that she has slept there for six consecutive years. The first impression is emphasised as the inspection proceeds, and ward after ward is visited. If we had to describe the Sussex County in a word, we should say it is essentially a comfortable hospital for patients, at any rate, though the smallness of the rooms and the necessity of placing the night nurses in a single dormitory would make it necessary to qualify this word before applying it to the staff.

The buildings at Brighton are old, the hospital having been opened in 1828. It is interesting to compare it with another old building, that of the Royal Infirmary, Bristol, which was erected nearly one hundred years earlier. At both institutions the management have taken infinite pains to do everything in their power to render the buildings suitable to the treatment of the sick. Taken together, they show the advance which was made in hospital construction during the century in question. At Bristol the wards are relatively small, and everywhere there is evidence that at the time the Royal Infirmary was built the importance of superficial space was not sufficiently appreciated. At Brighton, on the other hand, the superficial space is more than abundant, and the beds in each ward are able to be so arranged that day-room space for convalescent patients is easily provided. In both institutions the wards show that at the period when they were built the importance of cross ventilation was not only not appreciated, but not even understood. In the Sussex County the furniture, though scanty, has been selected with a view to the comfort of the patients, and the beds have been raised so as to enable the patients to look out of the windows, from some of which there is a good view. We would suggest the addition of a large and comfortable couch or seat to each of the large wards, of a pattern similar to that often met with in asylums, a plan of which we hope to publish shortly.

### Recent Criticisms.

The Sussex County Hospital has been subjected to severe criticism by "An Ex-Governor," who at first wrote anonymously, and now avows himself as Mr. James Fair, of King's Gardens, West Brighton. This gentleman's criticisms have been dealt with already in these columns. It is pleasant, however, to be able to record that refreshments are now provided for the out-patients; that the chapel is properly heated; that the charge of dreariness of aspect certainly does not apply to the wards; that the corridors do not need any further system of warming, such as Mr. Fair demanded; and that a site has been found for the lift, which will be erected as soon as funds are forthcoming to enable the committee to defray the cost. As we have already pointed out, the day-room space in the wards is abundant, and there would certainly be no justification for expending money in the provision of sitting-room accommodation for the patients. It will thus be seen that "Ex-Governor" has nothing left to criticise, except expenditure. Let us proceed to examine this in detail.

### The Expenditure.

We certainly think the Board would be well advised to appoint a small Committee to consider the question of expenditure. A careful consideration of the figures published in the reports in comparison with those published by other institutions fairly comparable with the Sussex County Hospital shows that the expenditure at the latter is undoubtedly higher than elsewhere. This may be due to the absence of contracts, to the paying of higher prices for various articles, or to some cause unknown. It is partly accounted for, we believe, by the fact that the proportion of in-patients to each bed occupied is only from eight or nine to one, as compared with thirteen or fourteen to one in institutions like the Liverpool Northern Hospital and the Hull General Infirmary. It has been maintained, though, as we have shown on more than one occasion, with probable small reason, that a comparison between the expenditure of various institutions should be for a term of years rather than for a particular year. We have not space to give the figures for a series of years, though we have taken the trouble to get them out. We must, therefore, content ourselves with those for the year 1889. It happens that the Norfolk and Norwich County Hospital and the Sussex County Hospital are not only identical in having to minister to the needs of an agricultural and urban population, but that the number of beds occupied is usually almost identical at these two institutions. At Norwich the buildings are comparatively recent as compared with the old buildings at Brighton, but this fact cannot account for a difference in the expenditure of £18 per bed, the cost at Norwich in 1889 being £55 per bed, and at Brighton £73 per bed in round numbers. It will be seen from the table given below that the expenditure at Brighton is higher under nearly every sub-head, a fact which should engage the immediate and earnest attention not only of the Committee but of the medical staff. In 1889 the Norfolk and Norwich Hospital contained 200 beds, and the average daily number of beds occupied was 138. The number of in-patients under treatment was 1,280; the number of out-patients 4,302; and the proportion of in-patients to each bed occupied, 9 to 1. At the Sussex County Hospital, in 1889, there were 173 beds, of which 138 were daily occupied. The number of in-patients was 1,259, and of out-patients 5,170; the proportion of in-patients to each bed occupied being 9 to 1. If the system pursued had been the same the cost per bed ought to be practically identical. The following table shows the expenditure per occupied bed in the two institutions, under various heads:—

| Items in 1889. | Sussex County Hospital. £ | s. | d. | | Norfolk and Norwich Hospital. £ | s. | d. |
|---|---|---|---|---|---|---|---|
| Provisions .. ... ... ... | 27 | 9 | 4 | ... | 19 | 16 | 5 |
| Domestic expenses, including gas, | 2 | 5 | 7 | ... | 1 | 15 | 9 |
| coal, firing, washing, bedding, linen, &c. ... ... ... ... | 9 | 10 | 5 | ... | 11 | 3 | 1 |
| Surgery and dispensary ... ... | 9 | 5 | 2 | ... | 4 | 1 | 10 |
| Salaries and wages... ... ... | 15 | 5 | 5 | ... | 12 | 10 | 11 |
| Pensions ... ... ... ... | 0 | 16 | 11 | ... | 0 | 4 | 2 |
| Repairs (ordinary) ... ... ... | 5 | 0 | 8 | ... | 0 | 19 | 3 |
| Incidental expenses ... ... | 3 | 1 | 11 | ... | 4 | 1 | 1 |
| Total cost per occupied bed in 1889 | £72 | 15 | 5 | | £54 | 12 | 6 |

The expenditure at the Sussex County Hospital is materially higher than that of the Norfolk and Norwich Hospital on every item except domestic and incidental expenses. The fact that the expenditure per occupied bed on alcohol is 10s. per bed higher at Brighton than at Norwich, ought to induce the medical staff to look into this question very closely, although we are glad to see that they have reduced this item by 10s. per occupied bed in the year 1889, as compared with the year 1888. There is also a reduction of £2 per bed compared with the previous year in domestic expenses, 12s. per bed in the salaries, and £1 5s. per bed in the item of repairs. Incidental expenses are also nearly 17s., and provisions nearly £2 per occupied bed lower in 1889 than in the previous year. Of course, it may be found on examination that both the quantity and the quality of the food supplied at Brighton is better than that at Norwich, though we can scarcely credit this, especially as the meat bill is £630, and the milk bill nearly £175 higher at Brighton than at Norwich, although the average daily number of beds occupied was 138 in 1889 at both institutions. It is surprising that drugs and other medical appliances, included under "Surgery and Dispensary," should have cost nearly £1,278 at Brighton, when the expenditure under this head only amounted to £565 in round numbers at Norwich. The causes of these differences, be they what they may, deserve the closest consideration, and we shall be interested to learn what is the explanation of them. It would surely be worth while for a small Committee of the Sussex County Hospital to visit Norwich with the view of clearing up the matter.

### THE NURSING HOME AND PENSION FUND.

There is one direction in which the Committee of the Sussex County Hospital ought to move at once. The accommodation for the nursing staff, taken as a whole, is not at present satisfactory, although the sitting-room and dining-room of the nurses are both comfortable and well found. Nurses are now supplied from the hospital to private cases, and the system has proved satisfactory and profitable. This being the case, the Committee would be well advised to communicate with the authorities of the National Pension Fund for Nurses with a view to adopting a scheme of affiliation, by which they would pay out of the profits made by the private nursing staff a sum equal to half the premiums of every nurse in their employ. We would further advocate steps being taken forthwith to erect a nurses' home and training school, where adequate sleeping accommodation, that is a separate bed-room for each nurse could be provided. In a wealthy town like Brighton, the sum required for this purpose must be relatively unappreciable, and we are certain that if asked for, the inhabitants would cheerfully supply it without hesitation or delay. In any case, whilst congratulating the committee upon the efficiency of its present resident staff and the ability and energy of the matron, Miss G. Scott, evidence of whose efficiency is manifested in every part of the hospital over which she exercises jurisdiction, we are bound to say that they will incur a very grave responsibility if they do not hasten to secure the erection of a Nurses' Home without delay.

It gave us great pleasure to inspect the Sussex County Hospital, and we are satisfied that those who are responsible for its management are anxious to leave nothing undone which will in any way tend to extend the usefulness and efficiency of its work, or to promote the well-being and speedy recovery of the patients treated within its walls.

